# The Topical Nanodelivery of Vismodegib Enhances Its Skin Penetration and Performance *In Vitro* While Reducing Its Toxicity *In Vivo*

**DOI:** 10.3390/pharmaceutics13020186

**Published:** 2021-02-01

**Authors:** Maria Natalia Calienni, Daniela Maza Vega, C. Facundo Temprana, María Cecilia Izquierdo, David E. Ybarra, Ezequiel Bernabeu, Marcela Moretton, Fernando C. Alvira, Diego Chiappetta, Silvia del Valle Alonso, María Jimena Prieto, Jorge Montanari

**Affiliations:** 1 Universidad Nacional de Quilmes, Departamento de Ciencia y Tecnología, Laboratorio de Bio-Nanotecnología, Bernal, Buenos Aires 1876, Argentina; natalia.calienni@unahur.edu.ar (M.N.C.); dmazavega@gmail.com (D.M.V.); cecilizq@gmail.com (M.C.I.); david.e.ybarra@gmail.com (D.E.Y.); fcalvira@gmail.com (F.C.A.); salonso@unq.edu.ar (S.d.V.A.); jime.prieto@gmail.com (M.J.P.); 2 Grupo de Biología Estructural y Biotecnología (GBEyB), IMBICE (CONICET CCT-La Plata), Buenos Aires 1906, Argentina; 3 Universidad Nacional de Hurlingham (UNAHUR), Hurlingham, Buenos Aires 1688, Argentina; 4 Universidad Nacional de Quilmes, Departamento de Ciencia y Tecnología, Laboratorio de Inmunología y Virología (LIV), Bernal, Buenos Aires 1876, Argentina; ctemprana@unq.edu.ar; 5 Consejo Nacional de Investigaciones Científicas y Técnicas (CONICET), Buenos Aires 1425, Argentina; eze_bernabeu@yahoo.com.ar (E.B.); marcelamoretton@gmail.com (M.M.); diegochiappetta@yahoo.com.ar (D.C.); 6 Universidad de Buenos Aires, Facultad de Farmacia y Bioquímica, Cátedra de Tecnología Farmacéutica I, Buenos Aires 1113, Argentina

**Keywords:** vismodegib, skin cancer, drug-delivery nanosystems, skin penetration, cytotoxicity, cellular uptake, zebrafish

## Abstract

Vismodegib is a first-in-class inhibitor for advanced basal cell carcinoma treatment. Its daily oral doses present a high distribution volume and several side effects. We evaluated its skin penetration loaded in diverse nanosystems as potential strategies to reduce side effects and drug quantities. Ultradeformable liposomes, ethosomes, colloidal liquid crystals, and dendrimers were able to transport Vismodegib to deep skin layers, while polymeric micelles failed at this. As lipidic systems were the most effective, we assessed the *in vitro* and *in vivo* toxicity of Vismodegib-loaded ultradeformable liposomes, apoptosis, and cellular uptake. Vismodegib emerges as a versatile drug that can be loaded in several delivery systems for topical application. These findings may be also useful for the consideration of topical delivery of other drugs with a low water solubility.

## 1. Introduction

Basal cell carcinoma (BCC) is the most common type of skin cancer, representing 80% of all cases [1]. Genetic alterations leading to the aberrant constitutive activation of the Hedgehog (Hh) signaling pathway, such as mutations which inactivate patched-1 tumor suppressor gene (PTCH1) or, less frequently, gain-of-function mutations in smoothened transmembrane protein (SMO), are associated with the development of most BCCs [2,3].

Vismodegib (Erivedge^®^, Genentech) is a first-in-class selective inhibitor for the SMO that acts on the Hh signaling pathway. Vismodegib was approved by the US Food and Drug Administration in 2012 for the treatment of locally advanced and metastatic BCC [4]. Currently, the treatment with Vismodegib consists of the daily oral administration of Erivedge^®^ capsules. However, there are several side effects associated with the systemic administration of this active principle that frequently cause patients to discontinue treatment [3].

An alternative to avoid these undesirable effects is to administer the active principle directly to the target site. Topical administration over skin cancer lesions appears as an interesting alternative to overcome side effects in healthy organs and tissues because of the local and site-specific application. Moreover, a site-specific application may allow the use of significantly lesser quantities of the drug than those required for systemic administration. However, Vismodegib presents a low skin penetration [5]. The access of the active principles to the skin is limited by the barrier of the stratum corneum (SC) [6] and by its physicochemical characteristics [7]. Recently, some efforts to administer Vismodegib locally have appeared in the literature, with the main objective of reducing such side effects and enhancing its skin penetration. Some of these consist of the use of ablative fractional lasers [8], microneedles [9], and micro- and nanoformulations for the topical delivery of the drug [5,10,11]. 

Particularly, nanomedicine provides tools to overcome the SC barrier to allow the targeted transport of actives by drug-delivery nanosystems. Five of the drug-delivery nanosystems reported to be used for topical application are ultradeformable liposomes (UDLs), ultradeformable ethosomes (UETs), colloidal liquid crystals, polyamidoamine (PAMAM) dendrimers, and polymeric micelles [10,12,13,14,15]. UDLs are highly flexible vesicles, composed of phospholipids and an edge activator, with an elastic energy in the order of the ambient thermal energy, which is almost 20 times lower than that of conventional liposomes. This feature gives them a high deformability that allows them to spontaneously cross the SC, driven by the force produced by the transdermal hydration gradient present in the skin [16], delivering both hydrophobic and hydrophilic drugs to deeper layers of the skin [17,18]. In recent years, the use of UDLs has been reported for the topical application of antitumor drugs [5,19,20,21]. UETs are flexible vesicles composed of phospholipids, an edge activator, ethanol, and water [22]. UETs are fluid systems in which ethanol acts as an enhancer of skin penetration [23]. These nanosystems have already been proposed for the topical treatment of skin cancer [24,25]. Colloidal liquid crystals, meanwhile, are nanocarriers composed of amphiphilic lipids that are able to self-assemble in a supramolecular structure [26]. Glyceryl monooleate is the main component of most of the topical formulations based on this technology that have been reported to date. There are some works that have used colloidal liquid crystals for the encapsulation of antitumors and skin delivery [26,27]. On the other hand, polymeric micelles are colloidal nanosystems formed by the self-assembly of amphiphilic copolymers above the critical micelle concentration which can be employed as nanocarriers of hydrophobic drugs [28,29,30], increasing their solubility in water. Recently, they also have been proposed for drug delivery through the skin [31,32]. Finally, PAMAM dendrimers G4 are nanomaterials with a highly branched macromolecular structure and amino-terminal groups on their surface. They are used as drug-delivery systems for diverse administration pathways, including the skin [33]. In all cases, the nanosystems proposed can encapsulate hydrophobic drugs such as Vismodegib [34].

This work aimed to compare the human skin penetration profile of different Vismodegib-loaded nanoformulations for topical application. Moreover, among these five nanoformulations we studied the cytotoxicity, cellular uptake, and induction of apoptosis of UDL-Vis, compared to the free drug, in two human cell lines that present the Hh pathway activated; the *in vivo* toxicity was also evaluated on zebrafish (*Danio rerio*) larvae. Recently, the zebrafish has been widely used as an intermediate model between *in vitro* determinations and *in vivo* studies with mammals because it is a growing model in the field of nanotoxicology [35].

## 2. Materials and Methods

### 2.1. Materials

Erivedge^®^ (Vismodegib) and Vismodegib standard (2-chloro-*N*-(4-chloro-3-(pyridin-2-yl)phenyl)-4-(methylsulfonyl)benzamide) were donated by Roche S.A.Q. e I. (Ricardo Rojas, Argentina). Soybean phosphatidylcholine and 23-(dipyrrometheneboron difluoride)-24-norcholesterol (TopFluor^®^ cholesterol) were purchased from Avanti^®^ Polar Lipids (Alabaster, AL, USA). PAMAM dendrimers G4 (ethylenediamine core), sodium cholate, Fluoroshield™ with DAPI, propidium iodide, Poloxamer^®^ 407, and Tween 80 were purchased from Sigma-Aldrich (Buenos Aires, Argentina). Glyceryl monooleate (Monomuls^®^ 90-O18) and Soluplus^®^ (polyvinylcaprolactam-polyvinyl acetate-polyethylene glycol graft copolymer) were purchased from BASF SE (Ludwigshafen, Germany). Chloroform, HPLC-grade trifluoroacetic acid, and acetonitrile were from J.T.Baker^®^ (Buenos Aires, Argentina). Crystal violet, neutral red, dimethyl sulfoxide, acetone, and methanol were from BioPack (Buenos Aires, Argentina). 3-[4,5-dimethylthiazol-2-yl]-3,5-diphenyltetrazolium bromide salt (MTT) was obtained from Life Technologies™ (Thermo Fisher Scientific Inc., Buenos Aires, Argentina). Sodium carboxymethylcellulose was from Fluka-BioChemika (Sigma-Aldrich, Buenos Aires, Argentina). Minimum essential medium, RPMI 1640 medium, fetal bovine serum, glutamine, pyruvate, penicillin, and streptomycin were obtained from Gibco (Waltham, MA, USA). All other reagents used were of analytical grade. 

### 2.2. Methods

#### 2.2.1. Purification of Vismodegib from Commercial Capsules 

Vismodegib was extracted from Erivedge^®^ capsules and measured as previously reported [5]. Briefly, the content of the capsules was extracted with methanol (1 mL of solvent to each 4 mg of Erivedge^®^) by vortexing for 1 min and then centrifuged to precipitate excipients. The supernatant was used for the subsequent studies. Reportedly, this method renders a 78.8 ± 7.2% recovery of Vismodegib. The purity and quantity of the drug were determined by RP-HPLC at 225 nm with a gradient of mobile phases containing trifluoroacetic acid and acetonitrile on a Waters Alliance 2690 liquid chromatography with a Waters Alliance 2487 UV-detector (Milford, MA, USA) and an Agilent ZORBAX Eclipse XDB-C18 column (150 × 3.0 mm, 3.5 µm particle size) (Santa Clara, CA, USA), using the Clarity 2.3 Software (DataApex, Prague, Czech Republic). A calibration curve with the standard was obtained (Y = 203.5 * X − 8.997; R^2^ = 0.9995) between 0.1 and 10 µg/mL by triplicate measurement.

#### 2.2.2. Obtention of Vismodegib-Loaded Nanosystems (UDL-Vis, UET-Vis, C-Vis, M-Vis, DG4-Vis)

Vismodegib-loaded ultradeformable liposomes (UDL-Vis) were prepared with soybean phosphatidylcholine (SPC), sodium cholate (NaChol), and Vismodegib in a mass ratio of 40:7:1.4, respectively, as reported in Calienni et al., 2019 [5]. Briefly, a thin lipid film was obtained and hydrated with 10 mM of Tris–HCl NaCl 0.9% *w*/*v* buffer, pH 7.4 (Tris buffer), obtaining a final SPC concentration of 40 mg/mL. Liposomes were extruded with a LipexTM 10 mL extruder (Transferra Nanosciences Inc., Burnaby, BC, Canada). Additionally, for *in vitro* assays with cell lines, some UDL-Vis were labeled (F-UDL-Vis) with TopFluor^®^ cholesterol, as a liposomal membrane label, and propidium iodide, as a label of the aqueous content. TopFluor^®^ cholesterol was co-solubilized with the lipids in a molar ratio of 1:500 fluorophore:SPC. The hydrophilic label propidium iodide (20 µM) was added to the resuspension buffer and, after the liposome obtention procedure, the non-encapsulated label was removed by gel permeation chromatography in a Sephadex G-50 column. No purification steps were needed in the case of TopFluor^®^ cholesterol, as it was totally incorporated into the liposome membranes.

Vismodegib-loaded ultradeformable ethosomes (UET-Vis) were prepared based on pre-existing methods [22,36] with ad hoc modifications. Briefly, SPC was dissolved in a solution of ethanol and Tween 80. Following this, Vismodegib was dissolved in the mix (SPC:drug mass ratio of 40:1.4) and then distilled water was added drop by drop under constant vortexing until opalescence. After water addition, the concentrations were SPC 2% *w*/*v*, ethanol 45% *w*/*v*, Tween 80 0.4% *w*/*v*. The suspension was stirred at 15,000 rpm for 1 min using a T-18 digital Ultraturrax^®^ (IKA-Werke GmbH & Co. KG, Staufen, Germany). Then, the excess ethanol was evaporated by stirring for 30 min at 700 rpm. 

Vismodegib-loaded colloidal liquid crystals (C-Vis) were prepared by dissolving Monomuls^®^ 90-O18 (90 mg) and Vismodegib (1.3 mg) in ethanol (2 mL), and dissolving Poloxamer^®^ 407 (50 mg) in distilled water (8 mL) [37]. The organic phase was dropped to the aqueous phase under stirring with T-18 digital Ultraturrax^®^. A total of three cycles of 5 min mixing at 18,500 rpm were carried out at room temperature. The suspension obtained was maintained at room temperature for 48 h under continuous stirring (400 rpm) to remove any trace of ethanol.

Vismodegib-loaded polymeric micelles (M-Vis) were prepared as follows: 10 mg of Vismodegib was dissolved in 2 mL of acetone and then the solution was added to a micellar dispersion of Soluplus^®^ 10% *w*/*v* in water (10 mL), as previously reported [38]. The sample was frozen (−20 °C) and lyophilized (48 h, condenser temperature of −40 °C and 0.03 mbar pressure; FIC-L05, FIC, Scientific Instrumental Manufacturing, Argentina). Then, the sample was resuspended in distilled water, allowing it to repose for 2 h at 4 °C before use.

Finally, Vismodegib-loaded PAMAM dendrimers G4 (DG4-Vis) were obtained by co-dissolving Vismodegib in methanol with PAMAM dendrimers G4 in a 1:2 × 10^−5^ Vismodegib: dendrimer molar ratio. The mix remained under stirring (150 rpm) for 24 h. Following this, the solvent was totally evaporated in a Savant Speed-Vac system AES 1010 (GMI, Inc., Ramsey, MN, USA) equipped with an RH 40-11 rotor under a vacuum for 2 h. Pellets were resuspended in Milli-Q water up to a final concentration of 1.3 mg/mL of Vismodegib. 

#### 2.2.3. Characterization of the Nanoformulations

Dynamic light scattering (DLS) was employed to assess the mean particle sizes of the diverse nanoformulations using a Nanozetasizer (Malvern, Malvern, UK). The Z-potential was determined with the same equipment.

Deformability was assessed for ultradeformable liposomes and ethosomes by adapting the method from van den Bergh et al., 2001 [39], evaluating the pass of the nanoformulation through a membrane of 50 nm-diameter pores under a pressure of 0.9 MPa of N_2_ in the LipexTM automatic extruder. For each formulation, 1 mL of suspension was loaded, and then aliquots from the outlet were collected each 30 s following lipid determination by a colorimetric method [40]. Conventional liposomes were used as a control for a non-deformable nanoformulation. 

Aliquots from all formulations were negatively stained with phosphotungstic acid and observed by transmission electron microscopy (TEM) in a JEM 1200EX II (Jeol Ltd., Tokyo, Japan).

#### 2.2.4. Skin Penetration Assays

To evaluate the skin penetration of Vismodegib loaded into the obtained nanoformulations, the Saarbrücken Penetration Model (SPM) was employed [41,42]. Explants from aesthetic surgery discards of a healthy Caucasian female patient (38 years old) were employed. After fatty tissue removal, discs of 24 mm diameter containing the SC, viable epidermis, and dermis (VE+D) were obtained with a punch and mounted on the Teflon piece from the SPM with the SC facing upwards. At the bottom of the Teflon device, 200 µL of Tris buffer was added [43]. In all cases, a total of 50 µL of each formulation was added in droplets of 2.5 µL over the explants and incubated for 4 h at 35 °C. Experiments were performed in quadruplicate sets. In order to compare the mass of Vismodegib recovered from the skin, all the results were relativized to the same initial quantity of drug incubated.

After incubation, explants were attached to a polystyrene block with metallic pins and the SC was removed by the tape stripping technique, consisting of charging a piece of adhesive tape with 2 kg for 10 s over the explant, extracting one SC layer after tape removal. The procedure was repeated 20 times, clustering tapes in 3 groups, with tapes 1 to 5 corresponding to shallow SC, 6 to 10 corresponding to medium SC, and 11 to 20 corresponding to deep SC [44]. The remaining part of the explant (VE+D) was homogenized and collected in a fourth tube for each skin explant. After tape stripping, the extraction of Vismodegib from the obtained samples was performed as in previous reports [5] with 3 mL of DMSO at 190 rpm in a shaker for 1 h at 37 °C. The determination was carried out by RP-HPLC UV as stated in Section 2.2.1. Tapes and VE+D from non-incubated skin samples were used as control.

#### 2.2.5. *In Vitro* Studies on HaCaT and SK-Mel-28 Cell Lines

For the *in vitro* studies, two human-derived cell lines (Instituto Multidisciplinario de Biología Celular-CIC-CONICET, Tolosa, Argentina) were used: HaCaT, immortalized non-tumorigenic keratinocytes, and SK-Mel-28, melanoma-derived cell line. Both have the Hh signaling pathway activated [45,46]. For all experiments, HaCaTs were maintained in RPMI 1640, whereas SK-Mel-28 were maintained in MEM supplemented with 1 mM of sodium pyruvate. Both cell lines were at 37 °C with 5% CO_2_ and supplemented with 10% heat-inactivated fetal bovine serum (FBS), 2 mM glutamine, 100 IU/mL penicillin, 100 µg/mL streptomycin, and 0.25 µg/mL amphotericin B. 

##### Cytotoxicity Determinations 

Cell viability after 4 and 24 h of incubation with empty UDL, UDL-Vis, and free Vismodegib (solubilized in DMSO, with a final concentration of 1% of solvent in the well) was determined by three different methods: MTT, crystal violet (CV), and neutral red (NR).

A total of 1 × 10^4^ cells were seeded on each well of 96-well flat-bottom microplates. After 24 h, the medium was replaced with 100 µL of the different formulations diluted in cell medium with FBS. The Vismodegib concentration assessed ranged from 0.04 to 0.65 mg/mL, which correspond to concentrations of SPC from 1.6 to 25.8 mM. An untreated control with cell medium with FBS and a control with cell medium with FBS plus 1% DMSO were included. After incubation, the media were removed; cells were washed three times with phosphate-buffered saline pH 7.4 (PBS); and the MTT, CV, and NR assays were performed as described in Calienni et al., 2018 [19]. Measurements were performed with a cell imaging multi-mode reader Cytation 5 (BioTek Instruments, Winooski, VT, USA). The cell viability was calculated according to the following equation, where AbsT is the absorbance of treated cells and AbsC is the absorbance of the corresponding control (without or with 1% DMSO). Data were reported as the mean of three different experiments ± SD:

Cell viability (%) = (AbsT)/(AbsC) × 100.

##### Evaluation of Cell Apoptosis

The evaluation of the apoptotic induction was carried out with the Annexin V-FITC apoptosis detection kit (BD Pharmingen™, San Diego, CA, USA). Cells were seeded in 6-well plates at a density of 3 × 105 cells/well and allowed to grow for 24 h. The medium was then replaced with 2 mL of fresh medium containing UDL-Vis or free Vismodegib, the last with 1% DMSO, in a concentration corresponding to 0.32 mg/mL of Vismodegib. Cells were incubated for 4 h, washed with PBS, and trypsinized. An untreated control and a control with 1% DMSO were included. Three independent determinations were carried out for each condition. The staining was performed according to the kit instruction. A total of 1.9 × 10^4^ cells were analyzed within 1 h by flow cytometry (Becton Dickinson FACSCalibur, Franklin Lakes, NJ, USA), with FL1 and FL3 channels. Data were processed using BD CellQuest™ Pro 6.0 software (Becton Dickinson, Franklin Lakes, NJ, USA). 

##### Cellular Uptake

Evaluation of the Uptake by Flow Cytometry

The cellular uptake of F-UDL-Vis (0.32 mg/mL of Vismodegib) was monitored in HaCaT and SK-Mel-28 by flow cytometry. A total of 1 × 10^5^ cells were seeded per well in 24-well plates and allowed to grow for 24 h. On one hand, uptake kinetics was performed in duplicate to determine the optimal incubation time to carry out the study. For this, the cells were incubated with 400 µL of F-UDL-Vis diluted in culture medium for 1, 2, and 4 h at 37 °C. After the time, the cells were washed three times with PBS, trypsinized, and centrifuged at 125× *g*. The pellets were resuspended with 300 µL of PBS. Then, flow cytometry was performed to quantify the uptake of the F-UDL-Vis over time. The samples were excited with a 488 nm laser and the FL1 filter (530/30 nm) was used to detect TopFluor^®^ cholesterol. 

After determining the incubation time for both lines, cells were treated with the F-UDL-Vis diluted in culture medium at 4 and 37 °C. Cells and the nanoformulation were previously incubated at the corresponding temperature for 1 h before adding the sample. A total of 1.9 × 10^4^ cells were analyzed in duplicate by flow cytometry. Untreated controls were included in the study of kinetics and uptake at 4 and 37 °C.

As a control of the cell viability, the apoptosis detection test detailed in the section “Evaluation of cell apoptosis” was performed in parallel to untreated cells incubated under the same conditions.

Evaluation of the Uptake by Fluorescence Microscopy

Cell uptake was also monitored in both cell lines by fluorescence. Cells were grown for 24 h in 24-well plates and then incubated with 400 µL of F-UDL-Vis diluted in culture medium (0.32 mg/mL of Vismodegib) for 4 h at 4 and 37 °C. Before incubating, cells, as well as the nanoformulation, were maintained at the corresponding temperature for 1 h. Three washes with PBS were performed to eliminate the remnant formulation and cells were fixed with cold methanol for 1 min. After three washes with PBS, the nuclei of cells were stained with Fluoroshield™ with DAPI for 5 min and the samples were mounted with coverslips. Untreated controls, stained with DAPI, were included.

Microscopies were carried out using a Cytation 5 configured with DAPI, GFP, and RFP filter cubes, in combination with LED light sources (365, 465, and 523 nm, respectively), to detect DAPI, TopFluor^®^ cholesterol, and propidium iodide, respectively. The DAPI cube was configured with a 377/50 excitation filter and a 447/60 emission filter; the GFP cube used a 469/35 excitation and a 525/39 emission filters, and the RFP cube used a 531/40 excitation and 593/40 emission filters. Exposure settings were automatically determined for each color and were the same for all samples. The focus was set automatically using the DAPI signal as a reference. 

#### 2.2.6. *In Vivo* Toxicological Determinations

Studies were carried out on wild-type zebrafish (*Danio rerio*) larvae between 4 and 7 days post-fecundation (dpf). Adults were maintained and paired as described in Calienni et al., 2018 [47].

Three embryos of 1 dpf were placed in each well of a 96-well microplate containing 125 µL of E3 medium (NaCl 0.29 g/L, KCl 0.012 g/L, CaCl2 0.036 g/L and MgSO4 0.039 g/L in deionized water, and 50 ppb methylene blue). At 4 dpf, the medium was removed and replaced with 250 µL of serial dilutions in E3 of UDL-Vis and Vismodegib with 1% DMSO. Controls incubated only with E3 or E3 plus 1% DMSO were included. The concentration range of Vismodegib tested was 10.9–175 µg/mL for the free drug, whereas it was 40.6–650 µg/mL for UDL-Vis (1.6–25.8 mM of SPC). The treatment solution was not removed nor renewed during the study. Embryos and larvae were maintained at 28 ± 1 °C with a 14/10 h light/dark cycle up to the end of the assay.

Different toxicity endpoints were assessed in triplicate: the measurement of the swimming activity, the determination of alterations in heart rate, and morphological changes.

##### Swimming Activity 

The movement of larvae was recorded for 15 min at 4, 24, 48, and 72 h post-incubation (hpi) at room temperature with an automated device with a system of infrared detection (WMicrotracker, Designplus SRL, Buenos Aires, Argentina) [48]. Swimming activity was determined as the number of interruptions of the infrared microbeam arrangement and data were relativized to the control. A total of eight wells per condition were analyzed.

##### Heart Rate and Morphological Alterations

The heart rate and morphological changes were assessed at 72 hpi as described in Calienni et al., 2017 [49]. Eight larvae per condition were immobilized with sodium carboxymethylcellulose, photographed and a video was recorded with a Microsoft LifeCam Studio camera coupled to a trinocular microscope Nikon SMZ800 (Nikon Corporation, Tokyo, Japan). The number of beats over 15 s was counted and reported as beats/minute. The larval eye area, rostrocaudal length, spinal cord length, uninflated swim bladder, arched body, tissue ulceration, and pericardial edema were analyzed with the ImageJ software. 

#### 2.2.7. Ethics Statement

All the animal procedures were approved by the Institutional Committee for the Care and Use of Laboratory Animals from the National University of Quilmes (project identification codes: CICUAL-UNQ 013-15 from 07/14/2015 and CICUAL-UNQ 014-15 from 07/14/2015) (Buenos Aires, Argentina), and were performed in strict accordance with International Guidelines for Animal Care and Maintenance. Protocols involving human skin explants were approved by the Ethical Committee of the National University of Quilmes (project identification code: CE-UNQ N°1/2019, from 04/29/2019) (Buenos Aires, Argentina) and were in accordance with the Code of Ethics of the World Medical Association. 

#### 2.2.8. Statistical Analysis

Data obtained from *in vitro* experiments were analyzed using one-way ANOVA and multiple comparisons tests of Tukey, whereas *in vivo* assays were analyzed using one-way ANOVA and the multiple comparisons tests of Dunnett. In the last case, each sample was compared to the control. GraphPad Prism 6.0 (GraphPad Software Inc., San Diego, CA, USA) was used to conduct all statistical analyses. Only values with *p* < 0.05 were accepted as significant.

## 3. Results

### 3.1. Characterization of the Nanoformulations

**Figure 1:**
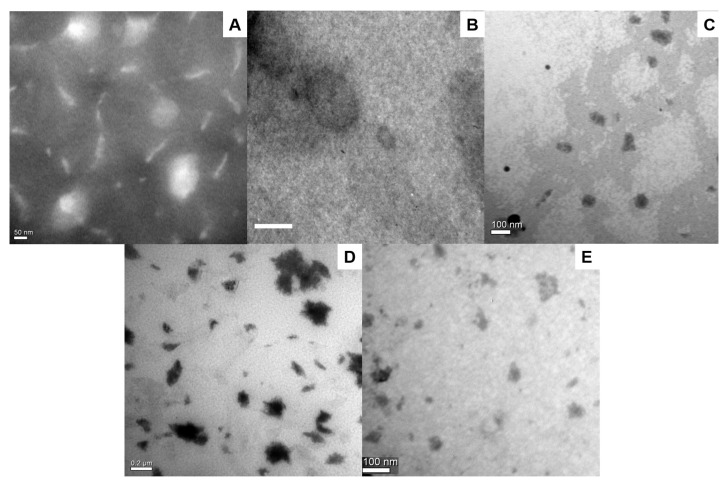
TEM micrograph of (**A**) UET-Vis (150,000×), (**B**) UDL-Vis (50,000×), (**C**) C-Vis (80,000×), (**D**) M-Vis (60,000×), and (**E**) DG4-Vis (100,000×). The reference bar corresponds to (**A**) 50 nm, (**B**) 100 nm, (**C**) 100 nm, (**D**) 200 nm, and (**E**) 100 nm. TEM micrograph of UDL-Vis can be found in reference [5].

The sizes and Z-potentials of the different Vismodegib-loaded nanoformulations are shown in Table 1. All the populations were in the expected range of mean size, with low polydispersity for UDL-Vis and UET-Vis. These populations also presented high-module values of Z-potential and aggregation was not observed in TEM micrographs (Figure 1A,B). UDL-Vis have been reported to render monodisperse populations with unilamellar structure [5]. In the case of M-Vis, polydispersity was high, while the Z-potential was very close to zero; these data are in concordance with TEM images (Figure 1D) in which M-Vis showed aggregation. On the other hand, C-Vis also presented a high polydispersity, but its Z-potential showed a higher value in the module than M-Vis. These parameters are in agreement with other works that obtained similar colloidal liquid crystals [50]. In this case, aggregation was not found in TEM images (Figure 1C). Finally, DG4-Vis could not be efficiently measured by DLS, while TEM images also showed some aggregation (Figure 1E).

No precipitate was observed after the obtention of UET-Vis, C-Vis, M-Vis, and DG4-Vis, leading us to conclude that all the drug had been incorporated into the nanoformulation because of the extremely low solubility of Vismodegib in water and the nature of the obtention methods. The loading efficiency of UET-Vis, C-Vis, M-Vis, and DG4-Vis was 0.17%, 0.92%, 0.99%, and 99.93%, respectively. For the case of UDL-Vis, the method rendered liposomes with an encapsulation efficiency of 91.98% ± 5.14 and a loading efficiency of 2.70%, as reported by Calienni et al., 2019 [5].

**Figure 2:**
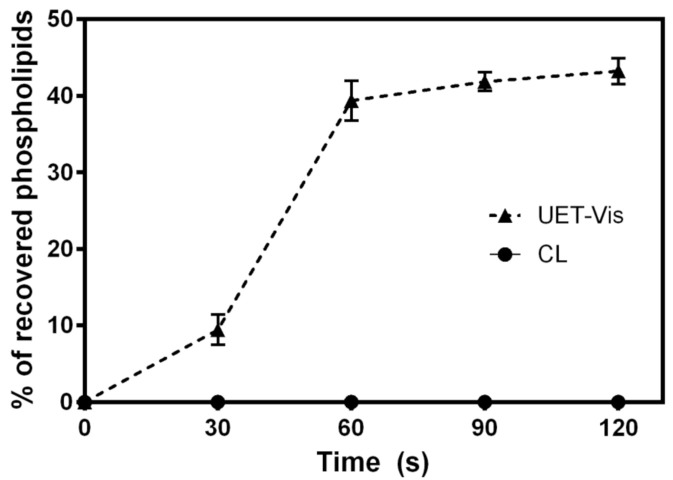
Profile of phospholipids’ passage through the 50 nm pore size vs. time to determine the deformability of UET-Vis. Conventional liposomes (CL) were used as a control for non-deformable formulation. Data are shown as mean ± SD (*n* = 3).

Since deformability is a key factor in the ability of UDL and UET to penetrate into deep layers of the skin, a deformability test was carried out for the nanoformulations loaded with Vismodegib. This assay allows one to determine if the ultradeformable formulations are capable of passing through pores with a mean size much smaller than their own size under low external pressure. It is critical to determine if the deformability was altered after the incorporation of a hydrophobic drug such as Vismodegib, because it is inserted into the liposomal membrane. For UET-Vis, the incorporation of the drug did not affect the deformability of the system. This can be seen in the deformability test in Figure 2, in which the formulation rapidly trespasses the nanopore, which is smaller than the mean diameter of the ethosomes, while a more rigid structure similar in size remains retained. The deformability test for UDL-Vis can be found in the literature [5], with similar results.

### 3.2. Skin Penetration

**Figure 3:**
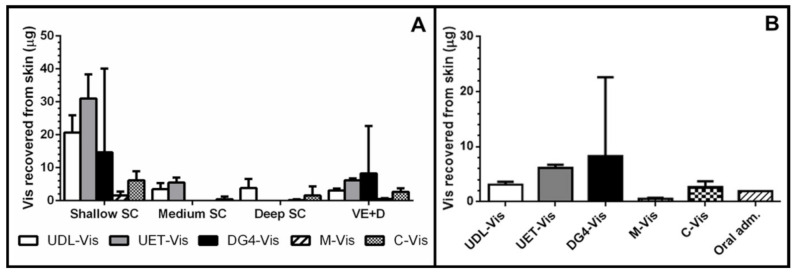
Human skin penetration. (**A**) Skin penetration profile of Vismodegib encapsulated in different drug-delivery nanosystems after 4 h of incubation on SPM. Mass of Vismodegib recovered from the shallow (tapes 1–5), medium (tapes 6–10), and deep (tapes 11–20) stratum corneum (SC) and viable epidermis + dermis (VE+D) are shown as mean ± SD (*n* = 4). (**B**) Mass of Vismodegib recovered from the VE+D after 4 h of incubation with the different nanosystems compared to the theoretical mass of Vismodegib reached by conventional oral administration. The amount of Vismodegib yielded after the conventional administration was obtained from previous work [5]. Data are shown as mean ± SD (*n* = 4).

As observed in previous works, Vismodegib cannot penetrate the SC of human skin per se, even when it is incubated with a penetration enhancer such as DMSO [5]. For this reason, it was encapsulated into different drug-delivery nanosystems that had shown skin penetration behavior. The skin penetration profile of Vismodegib encapsulated in each nanosystem was determined after 4 h of incubation (Figure 3A). 

The incorporation of Vismodegib into ultradeformable liposomes and ethosomes, dendrimers G4, and colloidal liquid crystals allowed the drug to penetrate the skin and to arrive in the VE+D, where neoplastic events develop. However, the micelles did not increase its skin penetration under the tested conditions, leaving only a small quantity of the drug in the shallow SC.

Given that Vismodegib needs to reach the VE+D to inhibit abnormal cells, the quantity of drug accumulated in this layer was compared between the different vehicles and with the estimated maximum mass of drug that arrives after oral administration in the same volume of skin −0.64 cm^3^ (Figure 3B). The concentration of Vismodegib in the skin after the conventional administration was calculated as ~3 µg/mL [5]. Results showed supratherapeutic delivery for most of the nanoformulations. UDL-Vis allowed the delivery of around 60% more drug after 4 h of incubation. For UET-Vis, this increased the amount of Vismodegib in the VE+D by three times, while the C-Vis delivery was slightly lower than that of UDL-Vis. On the other hand, DG4-Vis increased the mean amount of drug in VE+D, although it showed a high deviation and a lack of reproducibility. Finally, M-Vis, under these conditions, delivered less drug to the VE+D than the theoretical calculation for oral administration.

Under the tested conditions, the best results for skin penetration into the viable epidermis were obtained with the lipidic systems (UDL-Vis, UET-Vis, and C-Vis), which were specially designed for skin delivery. UDL-Vis were then chosen as a model lipidic formulation for further *in vitro* and *in vivo* studies.

### 3.3. In Vitro and In Vivo Studies

Cytotoxicity tests, the determination of apoptosis induction, and the liposome uptake were assessed on SK-Mel-28 and HaCaT, two human cell lines that present the Hh signaling pathway activated on which Vismodegib acts. Toxicity studies with zebrafish larvae allowed comparing the effects of UDL-Vis and free Vismodegib by analyzing some parameters considered toxicity endpoints.

#### 3.3.1. Cytotoxicity

Cytotoxicity assays consisted of the determination of metabolic activity by MTT, the integrity of membranes and lysosomes by NR, and the adhesion of cells by CV. The three trials were performed in triplicate to determine the cell viability after 4 and 24 h of incubation with UDL, UDL-Vis, and free Vismodegib. No significant differences were observed between the untreated control and the control with medium plus 1% DMSO. The three techniques yielded similar results for both cell lines. 

**Figure 4:**
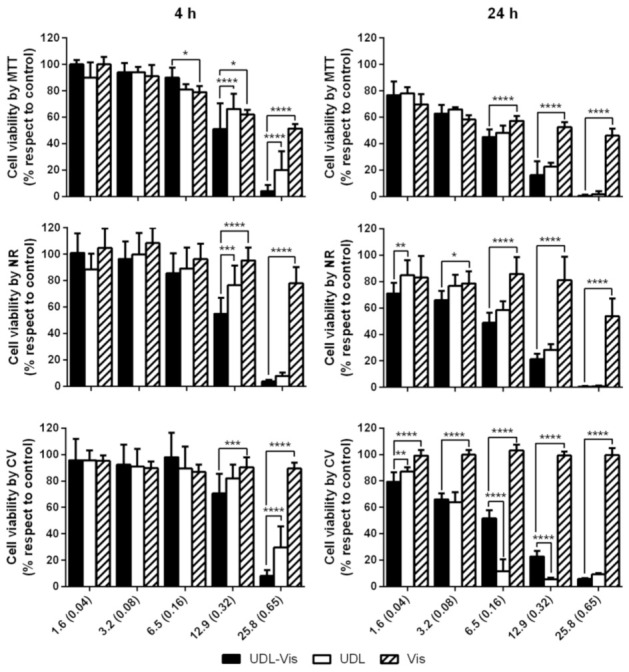
Percentage of cell viability of SK-Mel-28 after (**4 h**) and (**24 h**) of incubation with UDL, UDL-Vis, and free Vismodegib (Vis) as determined by MTT, neutral red (NR), and crystal violet (CV). Concentrations are expressed as the concentration of SPC in mM and within the parenthesis the concentration of Vismodegib in mg/mL. Data are presented as mean ± SD (*n* = 3). Statistical analysis is only shown in those concentrations in which the changes were significant with respect to the control (determined by one-way ANOVA analysis). * *p* < 0.05, ** *p* < 0.01, *** *p* < 0.001, **** *p* < 0.0001.

In the case of SK-Mel-28, the UDL-Vis was more cytotoxic than UDL and the free drug after 4 h of incubation (Figure 4) for the two highest concentrations studied (12.9 mM SPC-0.32 mg/mL Vismodegib and 25.8 mM SPC-0.65 mg/mL Vismodegib). UDL-Vis was also significantly more cytotoxic than the free drug after 24 h of incubation, even from lower concentrations. Only in the case of the CV test, it was observed that, at intermediate concentrations, the UDL affected the adhesion of the cells to a greater extent compared to the UDL-Vis. 

**Figure 5:**
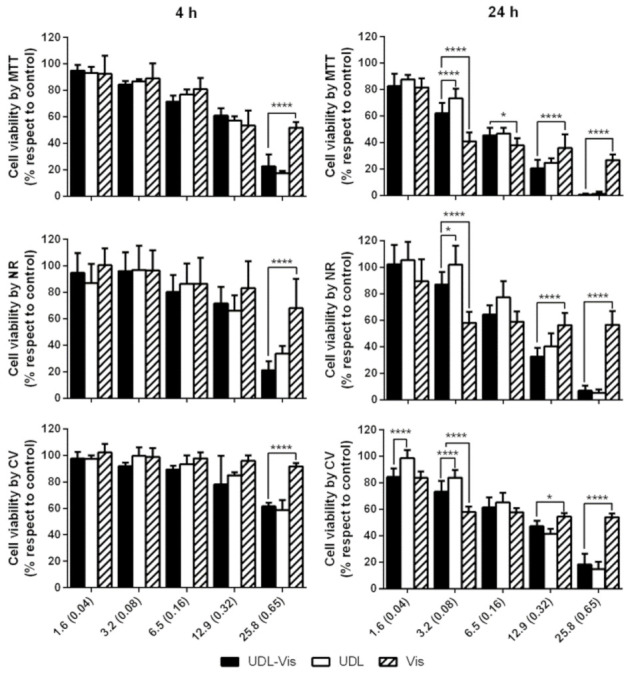
Percentage of cell viability of HaCaT after (**4 h**) and (**24 h**) of incubation with UDL, UDL-Vis, and free Vismodegib (Vis) as determined by MTT, neutral red (NR), and crystal violet (CV). Concentrations are expressed as the concentration of SPC in mM and within the parenthesis the concentration of Vismodegib in mg/mL. Data are presented as mean ± SD (*n* = 3). Statistical analysis is only shown in those concentrations in which the changes were significant with respect to the control (determined by one-way ANOVA analysis). * *p* < 0.05, **** p < 0.0001.

For HaCaT, after 4 h of incubation, only at the maximum concentration studied (25.8 mM SPC-0.65 mg/mL Vismodegib) was the UDL-Vis more cytotoxic than the free drug (Figure 5). After 24 h, UDL-Vis was more cytotoxic than Vismodegib for the two highest concentrations studied (12.9 mM SPC-0.32 mg/mL Vismodegib and 25.8 mM SPC-0.65 mg/mL Vismodegib). However, for lower concentrations, an inverse effect was observed.

#### 3.3.2. Apoptosis

**Figure 6:**
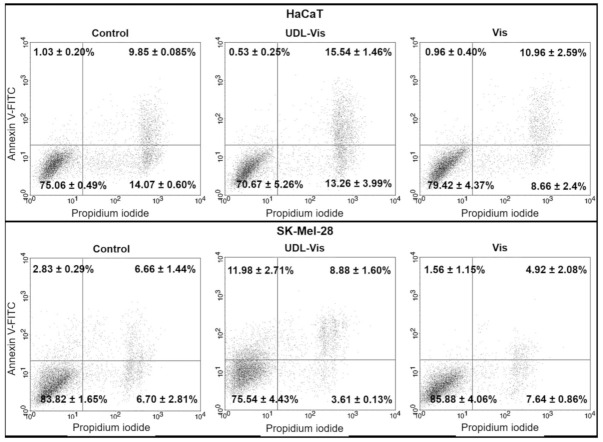
Detection and comparison of induction of apoptosis in (**HaCaT**) and (**SK-Mel-28**) by flow cytometry. Cells were analyzed after 4 h of incubation with free Vismodegib (Vis) and UDL-Vis. The events were detected using Annexin V-FITC and propidium iodide. The presented dot plots correspond to one of the three independent determinations, and the quadrants’ percentage values are the mean ± SD of those experiments (*n* = 3).

The induction of apoptosis was determined by flow cytometry after 4 h of treatment with the free drug and UDL-Vis using Annexin V-FITC and propidium iodide (Figure 6).

A differential effect was observed between both cell lines. In SK-Mel-28, the UDL-Vis induced a higher rate of apoptosis than the free drug. UDL-Vis also produced a marked increase in the number of double-labeled cells with respect to the free Vismodegib and the control, which could be in late apoptosis, undergoing necrosis, or already dead.

On the other hand, no considerable changes were observed in the induction of apoptosis in HaCaT, but an increase in the number of double-labeled cells when incubated with free Vismodegib and UDL-Vis was detected. That increase after treatment with UDL-Vis was greater than that with treatment with the free drug.

No differences were detected between the control incubated with or without 1% DMSO.

#### 3.3.3. Cellular Uptake

**Figure 7:**
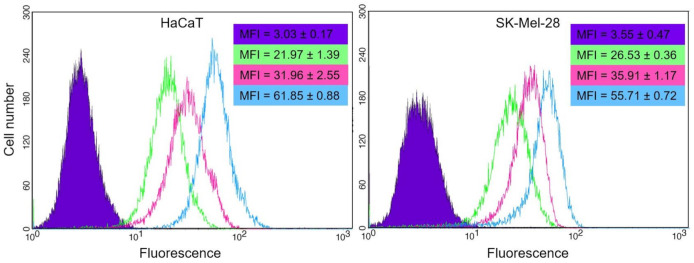
Histograms for (**HaCaT**) and (**SK-Mel-28**) were obtained by flow cytometry after 1 (green), 2 (pink), and 4 (light blue) hours of incubation with F-UDL-Vis at 37 °C. The untreated control is shown in violet. The presented histograms correspond to one of the replicates, and the mean fluorescence intensity (MIF) ± SD values obtained are denoted following the same color code. MFI is shown as mean ± SD (*n* = 2).

The cellular uptake of the nanoformulation was studied in both cell lines by different techniques. Firstly, the kinetics of the F-UDL-Vis uptake was monitored by flow cytometry to determine the optimal incubation time (Figure 7). Based on the results obtained, it was decided to incubate the HaCaT for 4 h. Although at 1 h practically all the cells showed the label, the fluorescence intensity was higher at 4 h. Meanwhile, for SK-Mel-28 it was decided to incubate them for 1 h.

**Figure 8:**
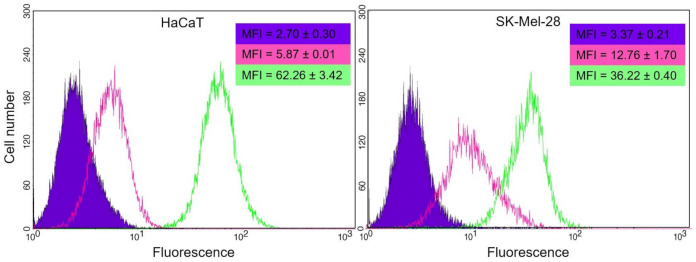
Histograms for (**HaCaT**) and (**SK-Mel-28**) were obtained by flow cytometry after incubation at 37 °C (green) and 4 °C (pink) with F-UDL-Vis for 4 h for HaCaT and 1 h for SK- Mel-28. The untreated control is shown in violet. The presented histograms correspond to one of the replicates, and the mean fluorescence intensity (MIF) ± SD values obtained are denoted following the same color code. MFI is shown as mean ± SD (*n* = 2).

To determine if the internalization of liposomes was mediated by metabolically active uptake, cells were incubated at 4 and 37 °C (Figure 8). In parallel, the viability of the controls was monitored under the same conditions of temperature and incubation time by means of the Annexin V-FITC apoptosis detection kit. There were no differences between the viability of controls at 4 and 37 °C for both lines.

In both cell lines, the uptake occurred mainly at 37 °C. However, a non-negligible level of fluorescence was observed after incubation at 4 °C for both, being notable in SK-Mel-28.

**Figure 9:**
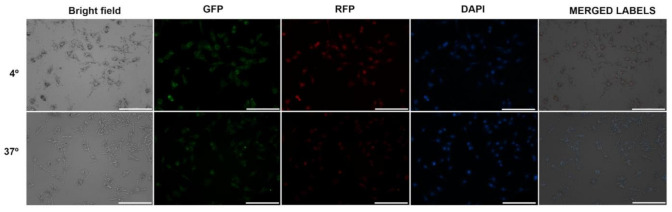
Microscopies (10×) of SK-Mel-28 cells after 4 h of incubation with F-UDL-Vis at 4 °C (upper images) and 37 °C (lower images). Green, red, and blue signals correspond to TopFluor^®^ cholesterol, propidium iodide, and DAPI, respectively. Filter cubes used to detect each fluorophore are indicated. Scale bar corresponds to 200 µm.

**Figure 10:**
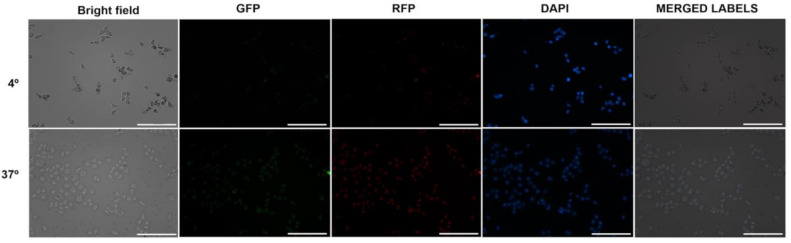
Microscopies (10×) of HaCaT cells after 4 h of incubation with F-UDL-Vis at 4 °C (upper images) and 37 °C (lower images). Green, red, and blue signals correspond to TopFluor^®^ cholesterol, propidium iodide, and DAPI, respectively. Filter cubes used to detect each fluorophore are indicated. Scale bar corresponds to 200 µm.

The internalization of F-UDL-Vis was also corroborated by fluorescence microscopy at 4 and 37 °C. In this case, two fluorophores were measured after 4 h of incubation in SK-Mel-28 (Figure 9) and HaCaT (Figure 10). TopFluor^®^ cholesterol was chosen as a liposomal membrane label and propidium iodide as a label of the aqueous content.

On one hand, there were observed TopFluor^®^ cholesterol and propidium iodide signals at 37 °C in both cell lines. On the other hand, both fluorophores were detected in SK-Mel-28 and HaCaT after incubation at 4 °C; however, the uptake at 4 °C was higher in SK-Mel-28, as determined by flow cytometry.

#### 3.3.4. *In Vivo* Studies

**Figure 11:**
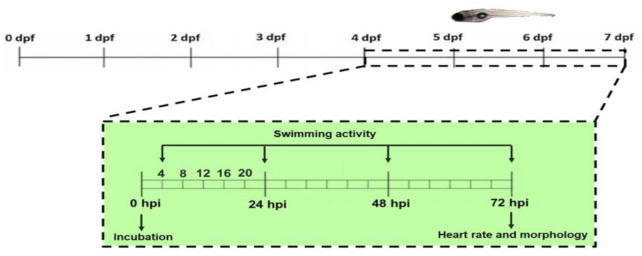
Timeline of zebrafish development in days post-fecundation (dpf). The inset shows the time in which determinations were carried out in dpf and hours post-incubation (hpi).

The toxicity endpoints that were analyzed were the following parameters: the determination of alterations in locomotor activity and heart rate and morphological changes (Figure 11). In all cases, no significant differences were observed between controls incubated with E3 medium and E3 medium with 1% DMSO.

##### Swimming Activity

**Figure 12:**
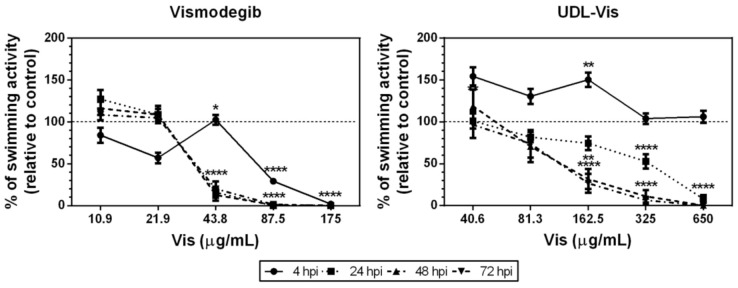
Percentage of spontaneous swimming activity of larvae treated with UDL-Vis and free Vismodegib with respect to the corresponding control (dotted line indicates 100% swimming activity), at 4, 24, 48, and 72 h post-incubation (hpi). Data are shown as mean ± SEM (*n* = 24). * *p* < 0.05, ** *p* < 0.01, **** *p* < 0.0001.

We determined the effect of UDL-Vis and free Vismodegib on the larval swimming activity, comparing it to that from the control at 4, 24, 48, and 72 h post-incubation (hpi) (Figure 12). The larval activity was measured by the automated WMicrotracker device, and the results were relativized to the control.

Free Vismodegib caused alterations in swimming activity at lower concentrations than the UDL-Vis did (Figure 12). The decrease in larval activity in both treatments at high concentrations could be related to the great mortality rate in the concentration ranges studied, with effects observed from 4 hpi in the case of the free drug. The UDL-Vis generated a decrease in larval activity with respect to the control that was time- and dose-dependent. At 4 hpi, incubation with UDL-Vis produced hyperactivity of the larvae at low concentrations which disappeared over time. This effect may be due to a transient initial excitation due to the presence of the nanosystem in the medium, as observed in other studies with nanoparticles [51]. 

##### Heart Rate Alteration and Morphological Changes

At 72 hpi, eight larvae per condition of the swimming activity studies were taken randomly. They were immobilized in sodium carboxymethylcellulose to be photographed and a 15 s video was recorded in order to count the beats/minute and determine the presence of morphological anomalies.

**Figure 13:**
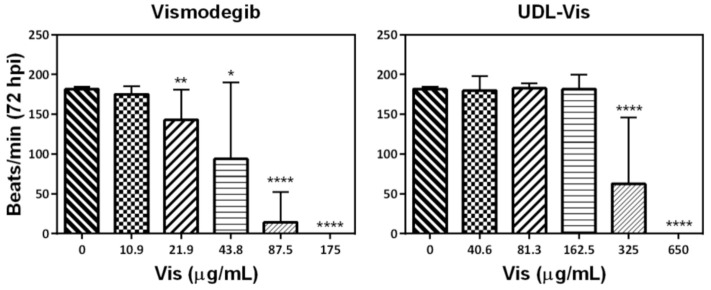
Heart rate of larvae at 72 h post-incubation (hpi) with free Vismodegib and UDL-Vis. Results are shown as mean ± SD (*n* = 8). * *p* < 0.05, ** *p* < 0.01, **** *p* < 0.0001.

One more time, it was observed that the free drug produced effects at lower concentrations than the UDL-Vis did (Figure 13). The larvae incubated with 175 µg/mL of free drug and 650 µg/mL of UDL-Vis were all dead after 72 hpi. 

Only one larva presented beats after incubation with 87.5 µg/mL of the free drug (it showed bradycardia), and only four larvae presented beats (one of them showed tachycardia) after incubation with 43.8 µg/mL of free Vismodegib. Besides this, larvae treated with 21.9 µg/mL of free Vismodegib presented mostly bradycardia. For the case of larvae treated with 325 µg/mL of UDL-Vis, only three larvae presented beats, and in all cases the heart rate corresponded to bradycardia.

On the other hand, since Vismodegib is a highly teratogenic drug, morphological alterations in the treated larvae were analyzed. The indicator parameters of teratogenesis analyzed were the presence of an uninflated swim bladder; arched body; tissue ulceration; pericardial edema; and changes in the eye area, rostrocaudal length, and spinal cord length.

**Figure 14:**
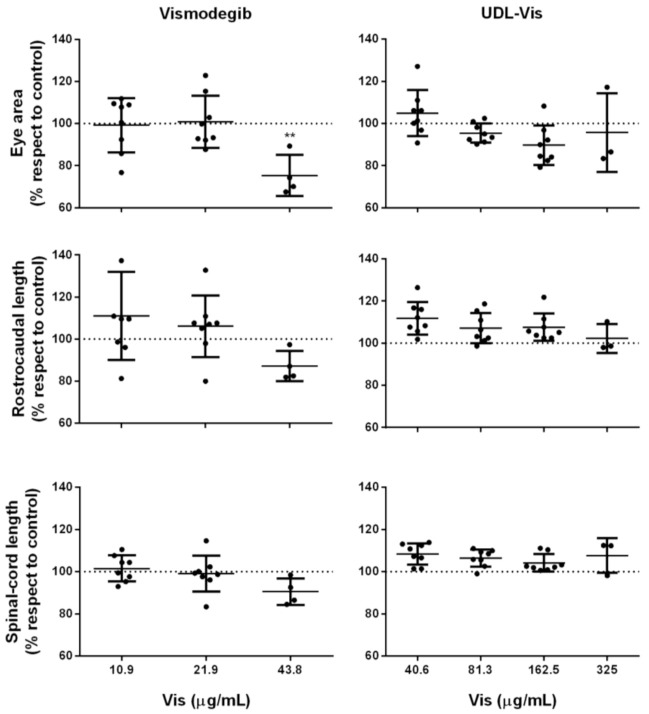
Morphological parameters were analyzed in larvae at 72 h post-incubation: eye area, rostrocaudal length, and spinal cord length. The measurements were carried out with the ImageJ software and were relativized to the corresponding control (dotted line corresponding to 100%). Results are shown as mean ± SD (*n* = 8), ** *p* < 0.01.

Only a significant decrease in the eye area was observed for the larvae treated with 43.8 µg/mL of free Vismodegib (Figure 14). In the concentration range studied, the UDL-Vis did not produce morphological changes. Larvae that were treated with concentrations greater than 325 µg/mL of UDL-Vis and 43.8 µg/mL of free Vismodegib could not be subjected to the morphological analysis, given their level of damage.

## 4. Discussion

This is the first work that compares the human skin penetration profile of different Vismodegib-loaded nanosystems suitable for topical application. Besides this, this is the first report of Vismodegib loaded into PAMAM dendrimers G4. The lipidic nanosystems achieved the best results, reaching a supratherapeutic concentration of Vismodegib in the VE+D, the site where BCC develops. The better skin penetration and delivery of the drug were expected for UDL, UET, and liquid crystals, due to the fact that they were specially designed for topical delivery. UDLs are specially designed for the skin delivery of drugs, with the presence of an edge activator (NaChol in this case) that drastically lowers their elastic modulus at room temperature, allowing them to penetrate the skin through the intercorneocytic pathway, impelled by the hydration gradient of the SC [17]. In the case of UET, Tween 80 is a non-ionic detergent that enhances the flexibility of membranes of the vesicles formed by SPC and ethanol in water. To evaluate deformability as a key factor for UDL and UET to penetrate deep layers of the skin, a test was carried out for these Vismodegib lipid-based nanoformulations. This can be seen in the deformability test in Figure 2, in which UET-Vis rapidly trespassed the nanopore, smaller than the mean diameter of the ethosomes, while a more rigid structure that was similar in size remains retained. In our previous work, a deformability test for UDL-Vis found similar results [5]. The ethanol present in UET-Vis increases the membrane fluidity and also enhances the cutaneous penetration of deep skin layers by temporarily disorganizing the SC layers [52]. In the case of the stable liquid crystalline nanostructures in the form of curved bicontinuous bilayers of lipid origin [26], they tend to accumulate in wrinkle-like defects of the SC [53] and in pilosebaceous follicles [13]. From there, Vismodegib in C-Vis can favorably enter the hydrophobic environment of the skin by partition. Another interaction that transiently impairs the barrier function of the SC and, in consequence, helps the entrance of loaded drugs into the skin is that of skin incubation with dendrimers [33]. Low-generation dendrimers have a good skin penetration profile and strong interactions between the dendrimer and the skin, particularly when their hydrophobicity is optimized through conjugation with hydrophobic molecules— [14] such as, in this case, Vismodegib. Although it was observed that DG4-Vis is agglomerated (Figure 1E), it was previously reported that cationic dendrimers alter the lipid layers of the skin, which could explain their penetration, as they can induce nanoscale holes in lipid bilayers [54]. Nevertheless, we observed a lack of reproducibility, which resulted in high SD values when the skin was incubated with DG4-Vis. An explanation of these differences between repetitions could be the aggregation of dendrimers, as this could be seen in the TEM images. However, it would be necessary to confirm the aggregation of the nanoformulation in aqueous suspension. Finally, unlike the other nanosystems, M-Vis delivered less drug to the VE+D than the oral administration. In the case of polymeric micelles, drug penetration depends on the rupture of the micelle in contact with the SC, as has been reported in a previous work [10]. Kandekar et al., 2019, obtained better results for Vismodegib in their system, remarkably with significantly longer incubation times, besides the differences in the matrix formulation [10]. With respect to the freeze-drying stage that was present in the M-Vis obtention process, we have previously found that it does not affect the size or Z-potential after rehydration [55,56], while we have also previously found that ultradeformable lipidic systems cannot be successfully rehydrated upon freeze-drying, suffering irreversible aggregation even with a high lyoprotectant content [57], and this was the reason why we choose to use only freshly prepared lipidic formulations in this study.

Due to lipidic nanosystems being the most effective, we chose UDL-Vis, with which we have previously been working [5], as a lipidic model nanoformulation to assess *in vitro* and *in vivo* toxicity. The encapsulation of Vismodegib into UDL increased its cytotoxicity compared to the free drug in SK-Mel-28 and HaCaT, both at 4 and 24 h of incubation. However, the melanoma-derived cell line (SK-Mel-28) was more sensitive to UDL-Vis than that derived from keratinocytes (HaCaT). Moreover, UDL-Vis was more cytotoxic than empty UDL, which could be a result of the synergy between the toxicity of the liposomal matrix, as has been observed in previous works [19,58], and that from the active principle. On the other hand, UDL-Vis would be internalized by the cells mainly by endocytic pathways, although its passive capture—not dependent on the metabolic activity at 37 °C—cannot be neglected. The uptake of liposomes at 4 °C was higher in SK-Mel-28 in relation to HaCaT, as observed by Calienni et al., 2018 [19], with 5-Fluorouracil-loaded UDL. Regarding the studies of apoptosis with Annexin V-FITC, the mechanism of death triggered in HaCaT could not be discerned, but it was observed that the encapsulation of Vismodegib in UDL increased cell death compared to the free drug. However, for SK-Mel-28 it was possible to determine that the UDL-Vis produced a significant increase in the induction rate of apoptosis compared to the same concentration of free Vismodegib.

Even though Vismodegib is the first drug specifically approved for the treatment of advanced basal cell carcinoma, SK-Mel-28 cells, which have the Hh pathway active, also responded favorably to the treatment with UDL-Vis. Therefore, it would be logical to think of the repositioning of Vismodegib for other types of skin cancer [59] and for other malignancies, such as pancreatic cancer [60], glioblastoma [61], and Gorlin syndrome [62], in which the Hh pathway is active. A similar potential versatility can be noted for UDL-Vis, which could be used to treat other skin cancers even in the first steps of the disease, potentially avoiding invasive therapies and surgeries, or could be used as adjuvant therapy. Because UDL allows the loading of both hydrophobic and hydrophilic drugs, it would be interesting to study the co-encapsulation of antineoplastic agents to obtain combined therapies for multiresistant tumors.

As for *in vivo* studies with zebrafish larvae, this is the first work to carry out these types of determinations with Vismodegib. Zebrafish is a growing model to test nanotoxicity that offers whole-animal information, which is impossible to obtain from *in vitro* studies, which can predict some adverse effects as a previous step to studies in mammals [63]. From the *in vivo* studies, it was observed that free Vismodegib was more toxic than UDL-Vis, since it caused alterations in swimming activity and heart rate at lower concentrations than UDL-Vis did. So, the encapsulation of the active principle in this nanoformulation would decrease its toxicity. Besides this, the concentration of Vismodegib (4.8 µg/mL) transported by UDL that reached the viable epidermis of human skin after 4 h of incubation was shown to be harmless to the larvae. In addition, the concentrations studied did not produce detectable teratogenic effects in the larvae, except for a mild to moderate effect shown in a single parameter in the treatment with free Vismodegib. 

## 5. Conclusions

The incorporation of Vismodegib in drug-delivery nanosystems suitable for the topical administration route could reduce the side effects reported after oral administration by site-specific delivery. Moreover, some nanosystems—UDL, UET, and colloidal liquid crystals—allowed reaching supratherapeutic concentration in the site of action while using lower quantities of the drug compared with the oral administration. Even though we have not studied if the drug can reach systemic distribution after topical administration, due to the amounts needed to be applied to the skin being significantly lower than those for the oral route, it would be expected not to see side effects or to see side effects drastically reduced. In the case of the PAMAM dendrimers G4, they were able to transport Vismodegib to deep skin layers, but there was no reproducibility of the assays, while the studied polymeric micelles failed at this.

Particularly, the incorporation of Vismodegib in UDL increased the cytotoxic effects of the drug in cells, as it activated the Hh signaling pathway and induced a higher rate of apoptosis than the free drug in a melanoma-derived cell line. UDL-Vis would be uptaken by the cells mainly by endocytic pathways, although passive internalization was also observed. On the other hand, this work brings new information about the toxicological effects of Vismodegib and UDL-Vis in the intermediate model zebrafish. These data are important for further studies in a murine model of basal cell carcinoma.

In summary, UDL-Vis could not only allow the topical delivery of the drug non-invasively in a concentration higher than that possible using the traditional route, but UDL could also enhance the performance of the active principle, possibly due to a synergic effect with the liposomal matrix. Vismodegib emerges as a versatile drug that can be loaded in several delivery nanosystems for topical application, and these findings may be also useful for the consideration of the topical delivery of other drugs with similar physicochemical characteristics.

## Figures and Tables

**Table 1:** Size and Z-Potential of Vismodegib-Loaded Nanoformulations.

Formulation	Size (nm)	PDI ^a^	Z-Pot. (mV)
UDL-Vis	116 ± 2	0.053	−19 ± 1
UET-Vis	130 ± 0.3	0.059	−15.2 ± 0.2
DG4-Vis	4–5 *	-	10.6 ± 1.7
M-Vis	132 ± 1	0.243	−0.103 ± 0.09
C-Vis	119.1 ± 1.8	0.218	−16.6 ± 1.7

^a^ Polydispersity index. * Manufacturer information.

## Data Availability

The data presented in this study are available on request from the corresponding author. The data are not publicly available due to privacy and ethicals.
